# Reflectance confocal microscopy mapping of nonmelanoma skin cancers to guide definitive radiation therapy: Case series

**DOI:** 10.1016/j.jdcr.2024.05.044

**Published:** 2024-08-24

**Authors:** Jack Madsen, Naiara Fraga-Braghiroli, Muni Rubens, Sreenija Yarlagadda, Noah S. Kalman

**Affiliations:** a Department of Radiation Oncology, Miami Cancer Institute, Baptist Health South Florida, Miami, Florida; b Department of Dermatology, Miami Cancer Institute, Baptist Health South Florida, Miami, Florida

**Keywords:** nonmelanoma skin cancers, radiation therapy, reflectance confocal microscopy

## Introduction

Basal cell carcinomas (BCCs) and squamous cell carcinomas (SCCs) are the most prevalent nonmelanoma skin cancer types. For patients with lesions in cosmetically challenging areas or for patients with significant comorbidities, nonsurgical management with radiation therapy can be an excellent alternative to surgery. As a surrogate for pathological margin assessment of surgical techniques, noninvasive tools to define the at-risk area can be extremely helpful when employing radiation to both preserve normal tissue from radiation side effects and to minimize the risk of local recurrence.

Reflectance confocal microscopy (RCM) allows for real-time, noninvasive “virtual-histologic” assessment of cutaneous neoplasms.^1^^,^^2^ Studies have demonstrated its value in defining surgical margins.^3^^,^^4^ Because of its success in presurgical mapping, our center started to utilize RCM for lesion margin mapping in patients receiving definitive radiation for BCC and SCC. Herein we present our 4-year experience with this pretreatment assessment for radiation margin delimitation. This retrospective 16-patient case series received IRB approval/exemption. Included patients had single clinical stage T1-2 lesions (American Joint Committee on Cancer version 8).

## Cases

**Fig 1:**
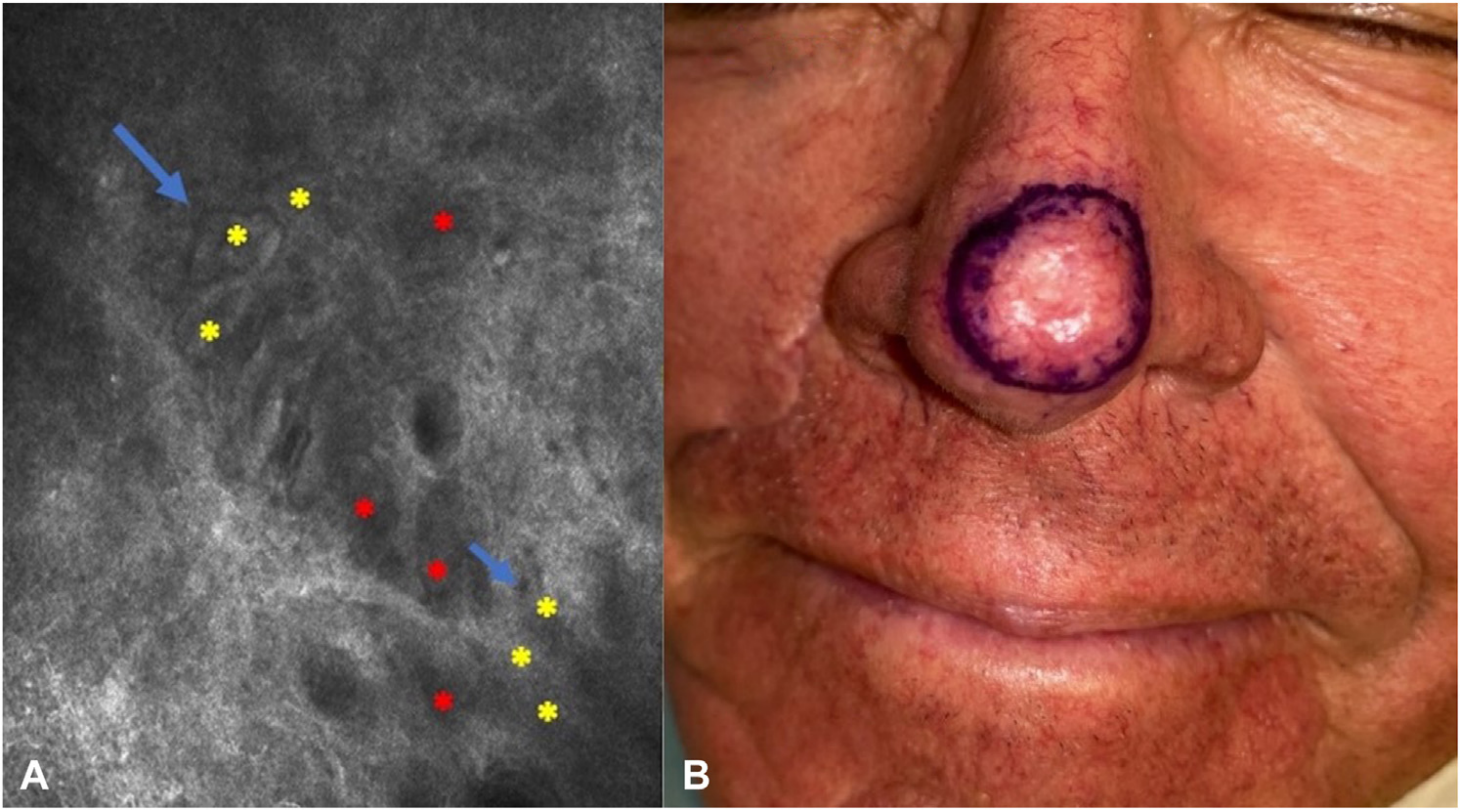
**A,** Reflectance confocal microscopy/Vivascope 3000 showing an oblique view of the spinous granular layer and dermal-epidermal junction (DEJ): dark silhouettes at the epidermal level (*red asterisks*) and small BCC tumor islands (*yellow asterisks*) surrounded by clefts (*blue arrow*) at the DEJ level. **B,** Clinical target delimited by RCM. *BCC*, Basal cell carcinoma; *RCM*, reflectance confocal microscopy.

All patients underwent dermatologic evaluation and RCM mapping with the Vivascope 3000 (hand-held probe) of the area to be treated immediately prior to radiation simulation (Fig 1, *A*). The radial lesion extent was marked and photographed. During the subsequent radiation simulation, the marked lesion border plus 0.5-1 cm was used to determine the radiation clinical target volume (Fig 1, *B*). After completion of radiation therapy, patients underwent routine follow-up.

Recorded clinical characteristics included histology (12 BCC and 4 SCC), radiation type (13 electrons, 3 superficial x-rays), dose fractionation (median 40 Gy in 8 fractions), and field size (median 39 cm^2^; range-9-250 cm^2^). All lesions were located in the face or pretibial regions, except for one patient with a peri-areolar BCC. Acute grade 2+ and 3 radiation dermatitis occurred in 47% and 18% of patients, respectively. Other grade 2+ acute toxicity was observed in 2 patients (fatigue and mucositis). For BCCs and SCCs, at median 12 and 13 month follow-up (range 2-52 and 6-40 months), respectively, no recurrences were seen.

## Discussion

This report demonstrates the feasibility and benefit of utilizing RCM (Vivascope 3000) to delimit treatment margins in patients receiving definitive radiation for nmelanoma skin cancers. Previous to employing RCM, radiation clinical target volumes had been determined by adding 1-2 cm to clinically visible disease to account for presence of subclinical disease. With limited follow-up, patients tolerated radiation well without local recurrence up to 4 years after the radiation. RCM may enable reduction of radiation field margins to reduce treatment toxicity while preserving local control.

## Conflicts of interest

None disclosed.
